# Compound heterozygous inheritance of two novel *COQ2* variants results in familial coenzyme Q deficiency

**DOI:** 10.1186/s13023-020-01600-8

**Published:** 2020-11-13

**Authors:** Aliaa H. Abdelhakim, Avinash V. Dharmadhikari, Sara D. Ragi, Jose Ronaldo Lima de Carvalho, Christine L. Xu, Amanda L. Thomas, Christie M. Buchovecky, Mahesh M. Mansukhani, Ali B. Naini, Jun Liao, Vaidehi Jobanputra, Irene H. Maumenee, Stephen H. Tsang

**Affiliations:** 1 Edward S. Harkness Eye Institute, Columbia University Irving Medical Center, New York, NY USA; 2 Laboratory of Personalized Genomic Medicine, Department of Pathology and Cell Biology, Columbia University Irving Medical Center, New York, NY USA; 3 Department of Ophthalmology, Hospital das Clínicas de Pernambuco (HCPE) - Empresa Brasileira de Serviços Hospitalares (EBSERH), Federal University of Pernambuco (UFPE), Recife, Pernambuco Brazil

**Keywords:** Coenzyme Q10, COQ2 gene, Oculorenal syndrome, Hereditary retinopathy

## Abstract

**Background:**

Primary coenzyme Q10 deficiency is a rare disease that results in diverse and variable clinical manifestations. Nephropathy, myopathy and neurologic involvement are commonly associated, however retinopathy has also been observed with certain pathogenic variants of genes in the coenzyme Q biosynthesis pathway. In this report, we describe a novel presentation of the disease that includes nephropathy and retinopathy without neurological involvement, and which is the result of a compound heterozygous state arising from the inheritance of two recessive potentially pathogenic variants, previously not described.

**Materials and methods:**

Retrospective report, with complete ophthalmic examination, multimodal imaging, electroretinography, and whole exome sequencing performed on a family with three affected siblings.

**Results:**

We show that affected individuals in the described family inherited two heterozygous variants of the *COQ2* gene, resulting in a frameshift variant in one allele, and a predicted deleterious missense variant in the second allele (c.288dupC,p.(Ala97Argfs*56) and c.376C > G,p.(Arg126Gly) respectively). Electroretinography results were consistent with rod-cone dystrophy in the affected individuals. All affected individuals in the family exhibited the characteristic retinopathy as well as end-stage nephropathy, without evidence of any neurological involvement.

**Conclusions:**

We identified two novel compound heterozygous variants of the *COQ2* gene that result in primary coenzyme Q deficiency. Targeted sequencing of coenzyme Q biosynthetic pathway genes may be useful in diagnosing oculorenal clinical presentations syndromes not explained by more well known syndromes (e.g., Senior-Loken and Bardet-Biedl syndromes).

## Introduction

Coenzyme Q10 (CoQ_10_), a key component of the oxidative phosphorylation pathway, is an essential factor in mitochondrial metabolism, and plays important roles in other cellular processes such as nucleotide synthesis, sulfide metabolism and cellular apoptosis. Endogenous defects in the biosynthesis pathway of coenzyme Q result in mitochondrial respiratory chain deficiency and associated manifestations. Involvement of the disease is systemic, and often includes neurologic manifestations (encephalopathy, hypotonia, cerebellar ataxia, progressive multiple-system atrophy like disease, spasticity, seizures, and intellectual dysfunction), kidney manifestations (steroid-resistant nephrotic syndrome and end-stage renal disease), hypertrophic cardiomyopathy, myopathy and sensorineural hearing loss [1, 2]. Ocular manifestations such as optic atrophy, nystagmus and retinopathy have also been described [1, 2]. Pathogenic variants in any of the genes involved in the biosynthetic pathway of coenzyme Q10 can result in a primary CoQ10 deficiency phenotype; in particular, the genes *COQ2* and *PDSS2* have been associated with retinopathy and optic atrophy, respectively. Depending on the severity of the variant and the deficiency, manifestations may be severe and appear in infancy or mild and begin in adulthood.

In this study, we identify two novel recessive variants in the *COQ2* gene which, when acting together in the compound heterozygous state, result in primary CoQ10 deficiency with associated nephropathy and retinopathy. In contrast to many other *COQ2* pathogenic variant phenotypes described, the phenotype here does not exhibit neurologic manifestations, but rather severe nephropathy combined with retinopathy in multiple affected members of the family.

## Materials and methods

### Subjects

A family with three affected siblings who presented to the Department of Ophthalmology at Edward S. Harkness Eye Institute, Columbia University was recruited and a complete ophthalmic examination along with multimodal imaging, electroretinography, and exome sequencing (ES) were conducted. Patient consent was obtained prior to genetic testing. All information in this report has been de-identified in accordance with HIPAA and institutional review board regulations.

### Ophthalmic Imaging

After measuring best corrected visual acuity, all patients’ eyes were dilated using topic 1% tropicamide and 2.5% phenylephrine followed by complete ophthalmic assessment including funduscopic examination, digital fundus photography, fundus short-wavelength autofluorescence (SW-AF) and spectral domain optical coherence tomography (SD-OCT). SW-AF (488 nm wavelength stimulus, barrier filtered transmitted light from 500 to 680 nm, 55° × 55° field) and SD-OCT images were taken by the use of a confocal scanning laser ophthalmoscope (cSLO; Spectralis HRA + OCT, Heidelberg Engineering, Heidelberg, Germany). SD-OCT images were acquired as horizontal 9 × 9 mm scans (870 nm light source and 7 μm axial resolution) positioned through the macula and obtained in high-resolution mode. The scans were recorded automatically by the use of recorded IR-R (820 nm light source) fundus images.

### Electroretinography

Full-field electroretinogram (ffERG) (Diagnosys, LLC, Lowell, Massachusetts, USA) was performed with Dawson Trick Litzkow (DTL) fiber electrodes and Ganzfield stimulation. ERG recordings were obtained for our patient for both eyes in accordance with the International Society for Clinical Electrophysiology of Vision (ISCEV) guidelines in both the scotopic and photopic states [3]. The amplitudes and implicit times recorded for each eye of the patient were compared to control patients with normal values and healthy eyes.

### Clinical exome sequencing (ES) and variant analysis

ES was performed on the three affected siblings at the Laboratory of Personalized Genomic Medicine (PGM) at Columbia University Irving Medical Center (CUIMC) on DNA obtained from peripheral blood. Written consent was obtained, exome sequencing libraries were prepared from genomic DNA from the probands using Agilent SureSelectXT (Human All Exon v.5 + UTRs) capture kit according to the manufacturers’ protocol. Paired-end sequencing was performed on the Illumina HiSeq 2500 platform. The sequence data were aligned and annotated using NextGENe (version 2.3.4.5; Softgenetics LLC, PA) software. Variant filtering and annotation were performed using an in-house developed proprietary analytical pipeline. Variants of interest were further analyzed using VarSome [4] and classified using the current ACMG/AMP guidelines [5].

## Results

### Clinical presentation

**Fig. 1:**
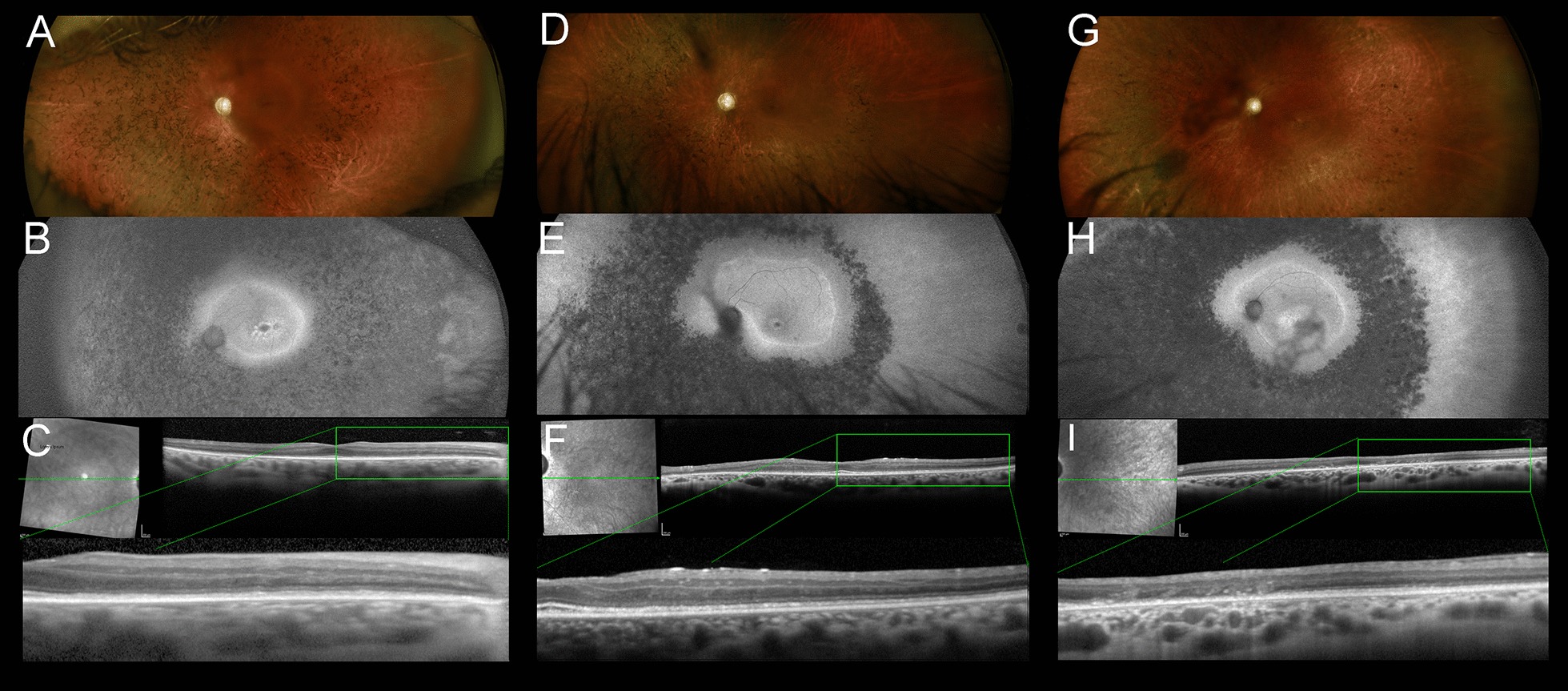
Multimodal Imaging of COQ2 Associated Rod-Cone Dystrophy. **a** Wide-field color fundus photographs demonstrating a pale optic nerve, attenuation of the vessels, and bony spicules in the periphery and midperiphery of the retina in the youngest proband. **b** Wide-field fundus autofluorescence images show hypoautofluorescence in the periphery with a ring of hyperautofluorescence in the macular region in the youngest proband. **c** Spectral-domain optical coherence tomography (SD-OCT) unveils retinal thinning due to atrophy of the outer retinal layers with regions of blurred or absent ellipsoid zone. Corresponding wide-field color fundus photographs, wide-field fundus autofluoresecence, and SD-OCT images showing similar findings in the affected brother (**d**–**f**), and affected sister (**g**–**I**)

A 25-year old man of Ashkenazi Jewish (AJ) descent presented to the clinic with new-onset difficulty with night vision. His medical history was notable for biopsy-proven diffuse glomerulosclerosis and associated hypertension. Immunofluorescence studies of his renal biopsy demonstrated no evidence supporting immune complex type disease, and the majority of glomeruli examined were globally sclerotic. Best-corrected visual acuity was 20/30 in the right eye (OD) and 20/25 in the left eye (OS), with no afferent pupillary defects. Visual fields to confrontation were constricted; anterior segment exam was unremarkable. His dilated fundus exam revealed healthy-appearing nerves and a flat normal-appearing macula, and clear vitreous in both eyes. Notably, he had extensive intraretinal pigment migration in the periphery arranged in a ring-like fashion, encircling the posterior poles in both eyes (Fig. 1a). Autofluorescence revealed hypoautofluorescence in a ring pattern mirroring the bone spicule pattern seen on clinical examination and color photography (Fig. 1b). Spectral domain optical coherence tomography (SD-OCT) revealed outer retinal atrophy in an annular pattern around the macula, with preserved outer retinal structure at the fovea (Fig. 1c).

The patient’s family history was notable for two of four other siblings with similar ophthalmic and medical histories. The parents showed no manifestation of disease and denied familial consanguinity. His first affected sibling was an older brother of 32 years of age, who was diagnosed with “retinitis pigmentosa” at age 21, and who had received a kidney transplant at age 5 years for a diagnosis of mesangial sclerosis. His medical history was also notable for elevated liver enzymes of unknown etiology and childhood acute lymphoblastic lymphoma that went into remission. On ophthalmologic exam, this sibling demonstrated a best corrected visual acuity of 20/25 OD, 20/30 OS, absence of relative afferent pupillary defects, and constricted visual fields to confrontation. Corneas were clear and the anterior segments were quiet, but he had a P1 posterior subcapsular cataract [6] in both eyes. Dilated fundus examination revealed nerves that were pale and cupped, flat normal-appearing maculae, and similar to the youngest sibling described above, peripheral ring-like patterns of bone speculation and pigment migration encircling the posterior poles in both eyes (Fig. 1d). Autofluorescence imaging showed hypoautofluorescent rings corresponding to the bone spiculated areas on color photography (Fig. 1e), and SD-OCT showed outer retinal atrophy in an annular pattern surrounding the macula, similar to his younger brother (Fig. 1f).

The patient’s second affected sibling was a 28-year old sister, also with a history of “retinitis pigmentosa” diagnosed at age 23, and a kidney transplant at age 10 for end-stage renal disease of unknown etiology; her birth history was notable for an extra digit on the right foot. Similar to her two affected siblings, her best-corrected central visual acuity was relatively good at 20/30 in both eyes (OU), without relative afferent pupillary defects. Her visual fields to confrontation were constricted, her corneas were clear and her anterior segments were quiet. Her lenses contained a P1 posterior subcapsular cataract [6] in both eyes. Her dilated fundus examination revealed healthy-appearing nerves, flat unremarkable maculae and clear vitreous. Again, her periphery was notable for extensive bone spiculation in a ring around the posterior pole OU, with mirroring autofluorescence patterns and outer retinal atrophy on SD-OCT (Fig. 1g–i).

**Fig. 2:**
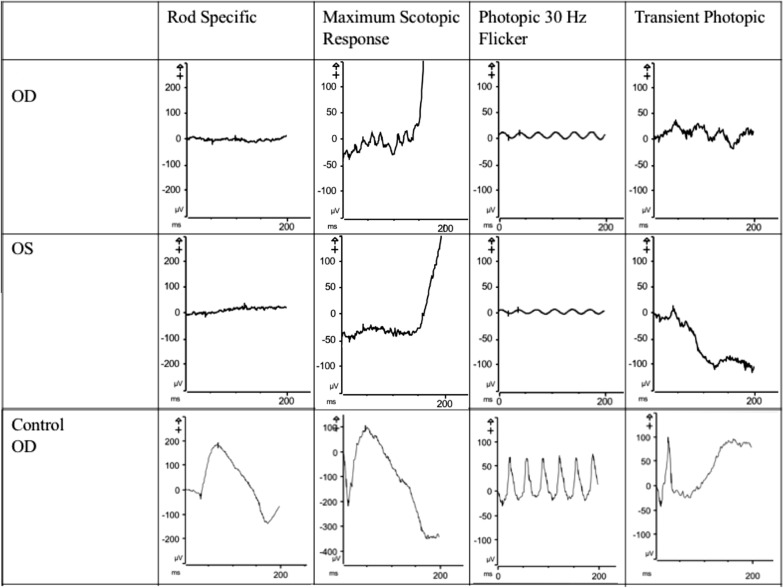
Electroretinography (ERG) testing. ERG testing for the youngest proband. Results shown here are representative of results obtained for all affected patients described in this report. Scotopic and photopic responses were extinguished in both eyes for all three patients, with scotopic responses being more severely affected, indicating a rod-cone dysfunction

Electroretinography (ERG) was performed on all three siblings and revealed a pattern consistent with rod-cone dystrophy, with an undetectable scotopic response (Fig. 2). All affected siblings had similar results with this modality of testing. An attempt was made to treat the underlying CoQ10 deficiency with exogenous coenzyme Q10 supplementation for all affected siblings for a period of 6 months to determine if any improvement could be detected on ERG readings (Tishcon Corp. Liposomal QH 10% ubiquinol solution, 10–30 mg/kg/day with the guidance of nephrology colleagues). Supplementation did not demonstrate any ERG improvement; however, both best corrected visual acuity and areas of retinal atrophy on autofluorescence were noted to be stable on treatment.

### Genetic analysis

**Fig. 3:**
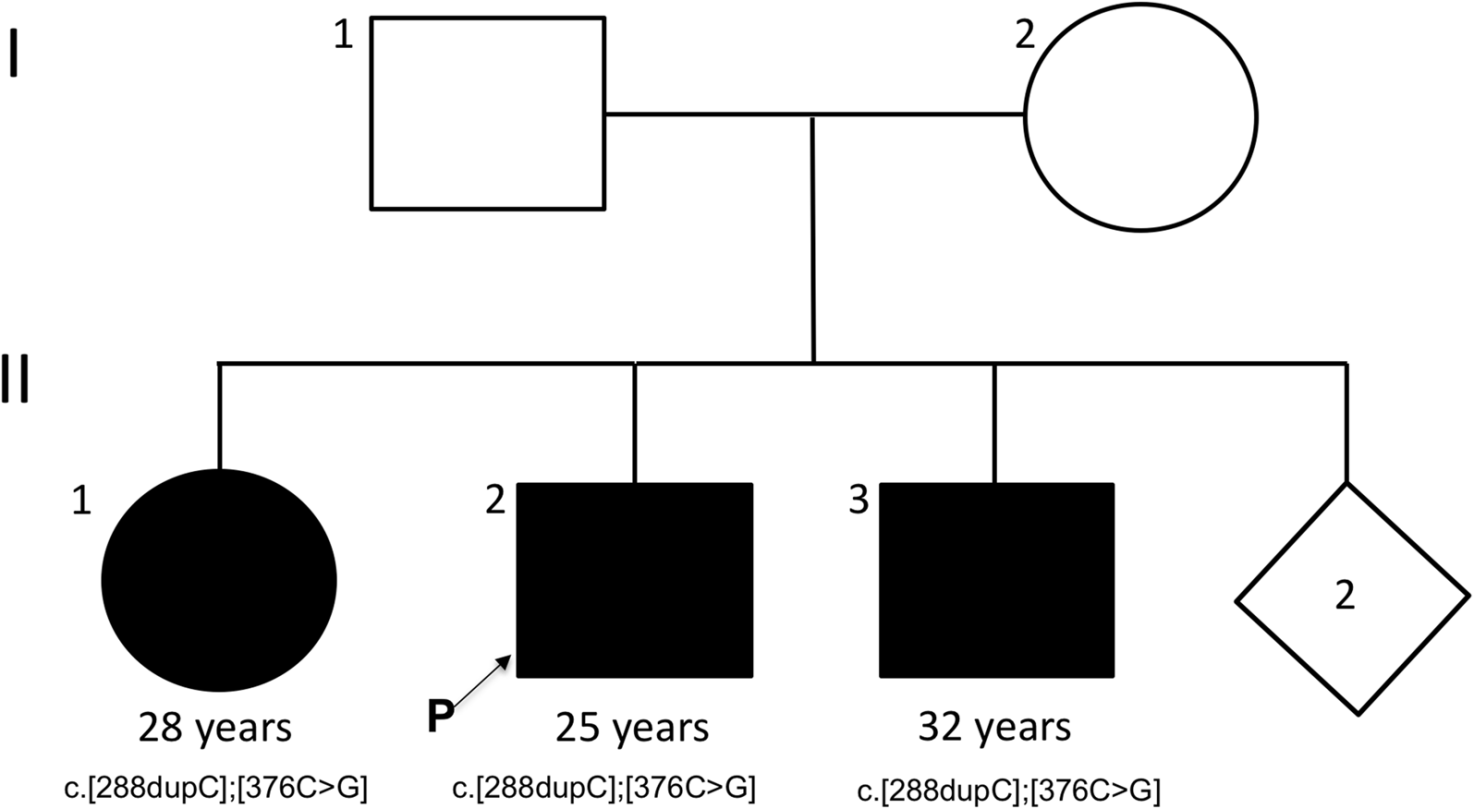
Family pedigree representation. Pedigree representation of affected family members. Segregation with disease of the variants in the *COQ2* gene in affected family members. Family pedigree member with arrow indicates the youngest proband; black symbol represents clinically affected individuals

ES was performed to determine the genetic cause of the three affected siblings’ phenotype. Although there was clinical suspicion for entities such as Senior-Loken syndrome 1–9 (MIM# 606996, 613615, 266900, 616629, 609254, 606995, 616307, and 610189) and Bardet-Biedl syndrome 1–21 (MIM# 209900, 615981, 600151, 615982, 615983, 605231, 615984, 615985, 615986, 615987, 615988, 615989, 615990, 615991, 615992, 615993, 615995, 615996, 617119, and 617406), no variants were detected in anyone of the corresponding genes; ES (see “Materials and methods” for detailed description) detected two heterozygous variants in the clinically relevant gene *COQ2* (NM_015697.8) that segregated in all affected individuals (Fig. 3).

**Fig. 4:**
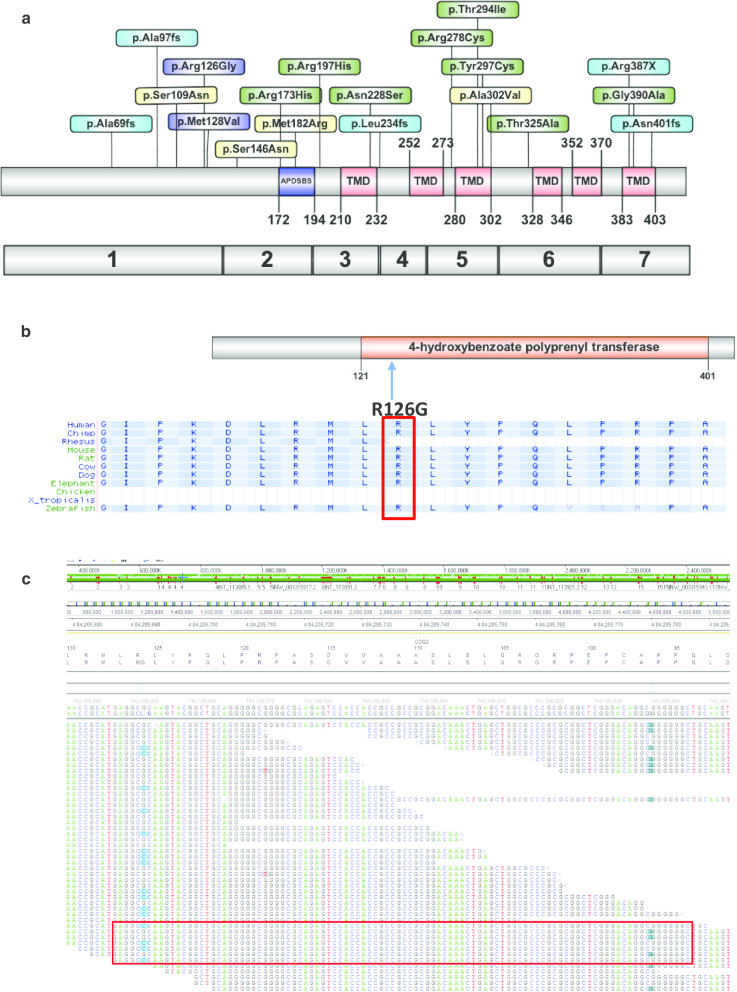
Genetic Results: Pedigree analysis, localization of *COQ2* variants, and next generation sequencing (NGS) pile-ups. **a** Localization of novel and reported *COQ2* variants in protein (NP_056512.5) and RNA (NM_015697.8) transcripts using the Illustrator for BioSequence (IBS) tool [30] Red boxes represent transmembrane domains per Forsgren et al. [31]; blue box represents an allylic polyprenyl diphosphate substrate-binding site (APDSBS); variants in light blue boxes are frameshift or non-sense variants; missense variants in yellow boxes are reported in patients with severe multisystem disease presentation; missense variants in green boxes are reported in patients with nephrotic syndrome with /without neurological symptoms; missense variants in purple boxes are reported in patients with multiple system atrophy; retinitis pigmentosa, and in the current study. **b** Conservation of p.Arg126Gly variant across multiple species and localization in the 4-hydroxybenzoate polyprenyl transferase domain. **c** NexGENe pile-ups demonstrating that the two *COQ2* variants are *in trans* in the youngest proband indicating compound heterozygous inheritance

The first variant was a duplication c.288dupC, in exon 1 of 7, which results in a frameshift and is predicted to cause premature termination of the protein, p.(Ala97Argfs*56).This variant was detected at an allele frequency of 6.987e-3 in gnomAD v3 exomes and genomes, seen in the heterozygous state in 10/143116 alleles, with no homozygotes. This suggests that it is not a common benign variant in the populations represented in these databases. A literature search revealed no previous patient reports described with this variant. There are conflicting predictions of pathogenicity for this variant in ClinVar (VarID: 631951), partly due to lack of reports in patients and higher allele frequency in the gnomAD Ashkenazi Jewish population (heterozygous in 8/3322 alleles (allele frequency = 0.2408, gnomAD v3). Though pathogenic frameshift variants reported in this gene are rare, there is one frameshift variant p.(Leu234Argfs*14) that terminates the protein downstream of this variant reported in the literature in a patient with isolated nephrotic syndrome (Fig. 4a). Based on current ACMG/AMP sequence variant interpretation guidelines, we classified this variant in our patients as pathogenic.

The second *COQ2* variant found in these patients, c.376C > G, p.(Arg126Gly) substitutes a glycine for an arginine at a conserved amino acid residue 126/422 in the hydroxybenzoate polyprenyl transferase domain of COQ2 (Fig. 4b) (Accession: TIGR01474, TIGRFAMS domain). In silico methods predicted this variant to be deleterious or damaging to protein function (Provean score: − 4.34 and SIFT score: 0.004). The variant is absent in gnomAD v3 exomes and genomes, which indicates that it is not a common benign variant in the populations represented in these databases. This variant is absent from ClinVar and no previously described cases were found in the literature. We classified this variant based on ACMG/AMP guidelines as a likely pathogenic variant.

Although neither parent in this family was tested for these two variants, clinical ES results revealed that the variants were present in different sequencing reads in the proband (Fig. 4c), indicating they came from different alleles and are in compound heterozygous state. This was possible as the variants are very close (88 bases apart) and phase could be determined based on the two variants not co-existing in the same sequence reads (100 bp in length). Similar results were also found in the older siblings, providing further evidence for bi-allelic inheritance of the two *COQ2* variants.

## Discussion

Here we describe two novel recessive potentially pathogenic variants: *COQ2*(NM_015697.8):c.288dupC,p.(Ala97Argfs*56) and *COQ2*(NM_015697.8):c.376C > G,p.(Arg126Gly), which together in the compound heterozygous state led to primary CoQ10 deficiency in three siblings. Due to limitations imposed by the COVID-19 pandemic, we were unable to confirm our genetic findings through measurement of CoQ10 levels from muscle biopsy or fibroblast samples. However, the pathogenicity of these variants is supported by several observations, including manifestation of symptoms in all three affected family members, population database analysis and in silico predictions. The combination of these variants in the compound heterozygous state primarily presented as severe progressive glomerulosclerosis leading to eventual kidney transplant at a young age, as well as progressive retinal degeneration mimicking retinitis pigmentosa.

Pathogenic variants of the *COQ2* gene and clinical manifestations, including neurologic and renal involvement, have previously been reported in the literature (Table 1). COQ2 deficiency has a variable presentation and can be associated with a severe multi organ system disorder [7–13], an intermediate nephrotic syndrome with or without neurological symptoms [9, 14–23], and milder later presentations of multi system atrophy and retinitis pigmentosa [24, 25]. The residual COQ2 function associated with a variant is shown to correlate with the severity of the phenotype [26]. Like previously reported cases, our patients exhibited renal degeneration, however they were unique in that that none of them developed neurologic abnormalities (but developed retinitis pigmentosa).

**Table 1:** *COQ2* variants and phenotypes published in the literature

References	Genotype:*COQ2* (NM_015697.8, NP_056512.5)	Phenotype	Effect of CoQ10 supplementation	Phenotype category
Diomedi-Camassei et al. [9]	c.[437G > A];[437G > A] p.[(Ser146Asn)];[(Ser146Asn)]	Acute renal failure, epileptic encephalopathy, hypotonia; died at 6 months	N/A	Severe multisystem
Mollet et al. [12]	c.[1197delT];[1197delT] p.[(Asn401Ilefs*15)];[(Asn401 Ilefs*15)]	Neurological distress at birth; liver failure and nephrotic syndrome at 2 days; died at day 12 from multi-organ failure	N/A	Severe multisystem
Jakobs et al. [11]	c.[905C > T];[905C > T] p.[(Ala302Val)];[(Ala302Val)]	Dizygotic twins; Premature birth, feeding problems, generalized edema, seizures, apnea; retinopathy of prematurity in one twin; infantile death	N/A	Severe multisystem
Scalais et al. [13]	c.[326G > A];[326G > A] p.[(Ser109Asn)];[(Ser109Asn)]	Myoclonic seizures, HCM, hypotonia, nystagmus (10 weeks);nephrotic syndrome (4 mo); died at 5 months	No improvement and continued deterioration	Severe multisystem
Dinwiddie et al. [8]	c.437G > A(;)1159C > T p.(Ser146Asn)(;)(Arg387Ter) Additional c.3754C > A variant in the *MT-ND1* gene	Neonatal diabetes, HCM, cardiomegaly, metabolic acidosis, encephalopathy, chronic renal tubular disease, respiratory failure, died at 2 months	N/A	Severe multisystem
Desbats et al. [7]	c.[545 T > G];[545 T > G] p.[(Met182Arg)];[(Met182Arg)]	Lactic acidosis, proteinuria, hepatic insufficiency, dilation of left ventricle, worsening neurological condition, died at 23 h	N/A	Severe multisystem
Eroglu et al. [10]	c.[437G > A];[437G > A] p.[(Ser146Asn)];[(Ser146Asn)]	Index case in family; neonatal diabetes, glomerular proteinuria, seizures, encephalopathy, hypotonia, electroretinography showed no response, death at 4.5 months	No improvement of renal or neurological symptoms	Severe multisystem
Eroglu et al. [10]	c.[437G > A];[437G > A] p.[(Ser146Asn)];[(Ser146Asn)]	Affected sibling; neonatal diabetes, proteinuria, mild metabolic acidosis, seizures, kidney failure, died due to multi-organ failure at 31 months	Normalization of glucose, lactate levels; neurological deterioration with encephalopathy and refractory seizures; deterioration of kidney function	Severe multisystem
Eroglu et al. [10]	c.[437G > A];[437G > A] p.[(Ser146Asn)];[(Ser146Asn)]	Index case in family; vomiting, proteinuria, nephrotic syndrome, refractory seizures, died at 6 months	N/A	Severe multisystem
Eroglu et al. [10]	c.[437G > A];[437G > A] p.[(Ser146Asn)];[(Ser146Asn)]	Affected sibling; neonatal diabetes, proteinuria, seizures, diffuse cerebral atrophy; died at 14 months	Proteinuria improved; continued neurological deterioration	Severe multisystem
Xu et al. [23]	c.[518G > A];[973A > G] p.[(Arg173His)];[(Thr325Ala)]	Edema, mild motor delay, moderate speech delay, nephrotic syndrome (11 months)	Edema resolved; improved kidney and motor development	NS + neurological symptoms
Quinzii et al. [19], Salviati et al. [20], Diomedi-Camassei et al. [9], Lopez-Martin [16], Montini et al. [18]	c.[890A > G];[890A > G] p.[(Tyr297Cys)];[(Tyr297Cys)]	Index case in family; epileptic encephalopathy, hypotonia, mild psychomotor delay, optic atrophy, nephropathy, rod-cone retinopathy (11 mo), kidney transplant (3 years)	Drastic improvement in neurological manifestations; no improvement in renal dysfunction	NS + neurological symptoms + RP
Quinzii et al. [19], Salviati et al. [20], Diomedi-Camassei et al. [9], Lopez-Martin [16], Montini et al. [18]	c.[890A > G];[890A > G] p.[(Tyr297Cys)];[(Tyr297Cys)]	Sister affected with only nephrotic syndrome (12 mo); no extra-renal involvement	Resolution of nephrotic syndrome	Isolated NS
Diomedi-Camassei et al. [9]	c.[590G > A];[683A > G] p.[(Arg197His)];[(Asn228Ser)]	Nephrotic syndrome (onset at 18 mo); no extrarenal involvement	Neurological examination continued to be normal after 8-mo of follow up	Isolated NS
McCarthy et al. [17]	c.[683A > G];[701delT] p.[(Asn228Ser)];[(Leu234Argfs*14)]	Nephrosis, renal failure (2 years)	N/A	Isolated NS
Bezdicka et al. [14]	c.[683A > G];[683A > G] p.[(Asn228Ser)];[(Asn228Ser)]	Two affected siblings; nephrotic syndrome (3 years)	Kidney function improved in sibling; no supplementation in index case	Isolated NS
Starr et al. [21]	c.[973A > G];[1159C > T] p.[(Thr325Ala)];[(Arg387Ter)]	Nephrotic syndrome (9 months)	Nephrotic syndrome resolved; continued proteinuria	Isolated NS
Starr et al. [21]	c.[176dupT];[683A > G] p.[(Ala69Argfs*33)]; [(Asn228Ser)]	Nephrotic syndrome (2 years)	Nephrotic syndrome resolved; continued proteinuria	Isolated NS
Wu et al. [22]	c.[832 T > C];[832 T > C] p.[(Cys278Arg)];[(Cys278Arg)] Also homozygous c.1213 + 1G > A variant in *ARSB* gene	Nephrotic syndrome, died (6 mo); no extrarenal involvement	N/A	Isolated NS
Starr et al. [21]	c.[683A > G];[881C > T] p.[(Asn228Ser)];[(Thr294Ile)]	Nephrotic syndrome (10 years)	Nephrotic syndrome not resolved, progressed to ESRD	Juvenile-onset NS
Gigante et al. [15]	c.[1169G > C];[1169G > C] p.[(Gly390Ala)];[(Gly390Ala)]	Index case in family with juvenile onset nephrotic syndrome and myoclonic epilepsy (16 years); affected cousin also with juvenile onset nephrotic syndrome (16 years), and headaches associated with phono-and photophobia	N/A	Juvenile-onset NS + neurological symptoms
Hara et al. [24], Mitsui et al. [25]	c.[382A > G];[382A > G] p.[(Met128Val)];[(Met128Val)] Additionally homozygous p.(Val393Ala) variant in *COQ2* also detected	Index case in family; multi-system atrophy (onset 68 years), RP (diagnosed at 33 years)	N/A	MSA + RP
Hara et al. [24], Mitsui et al. [25]	c.[382A > G];[382A > G] p.[(Met128Val)];[(Met128Val)] Additionally homozygous p.(Val393Ala) variant in *COQ2* also detected	Affected sibling; multi-system atrophy (onset 62 years),RP(diagnosed at 51 years)	N/A	MSA + RP
Current study	c.[288dupC];[376C > G] p.[(Ala97Argfs*56)]; [(Arg126Gly)]	Three affected siblings with renal abnormalities and RP	No improvement in ERG function	NS + RP

NS, nephrotic syndrome; ESRD, end-stage renal disease; MSA, multiple system atrophy; RP, retinitis pigmentosa

The *COQ2* gene was the first to be identified as a cause of primary CoQ10 deficiency when altered [19]. Although there are several reports of *COQ2* variants causing primary CoQ10 deficiency, only three of the reported patients with confirmed variants in the *COQ2* gene with associated retinopathy have been reported in the literature. The first patient was one of two affected siblings in a family who presented with nephrotic syndrome, neurological symptoms and rod-cone retinopathy [20]. His affected sister only presented with isolated nephrotic syndrome. Both siblings were homozygous for the variant, *COQ2* (NM_015697.8):c.890A > G,p.(Tyr297Cys). The other two patients were siblings with a homozygous variant, *COQ2*(NM_015697.8): c.328A > G, p.(Met128Val) that manifested as multiple-system atrophy and retinitis pigmentosa without clinical signs of nephropathy [27]. One of these siblings, similar to the patients in our series, was diagnosed with retinitis pigmentosa at a young adult age (33 years old), and developed neurological symptoms in the seventh decade of life. On post-mortem examination, her retina revealed severe loss of rods and cones and patchy disappearance of pigmentary epithelial cells [24]. The other sibling developed night blindness at the age of 48 and was subsequently diagnosed with retinitis pigmentosa. The siblings in our series differ from these aforementioned cases by presenting with severe nephropathy in addition to clinical retinitis pigmentosa, with absence of neurological involvement. The variants in our family are a combination of a damaging frameshift variant predicted to have no residual protein function and a missense variant. The intermediate phenotype seen in this family suggests that the p.(Arg126Gly) variant, similar to the neighboring p.(Met128Val), could be a mild variant with residual activity. Functional studies in yeast demonstrate that the p.(Met128Val) is a mild variant associated with residual activity [26], and therefore in the homozygous state leads to a late onset phenotype. Interestingly, both the p.(Met128Val) and p.(Arg126Gly) variants are found in patients with retinitis pigmentosa, suggesting that this region of the protein is likely essential for proper functioning of the protein in the eye. Although none of the siblings manifested any neurological abnormalities, we cannot exclude the possibility of neurological involvement later in life as with the previously reported cases.

Interestingly, *COQ2* is the only gene in the CoQ10 biosynthesis pathway that has been definitively linked with a retinitis pigmentosa-like presentation in the reported literature. There are two additional reports of patients with severe CoQ10 deficiency and retinopathy without confirmed variants in any of the biosynthetic pathway genes; rather, CoQ10 deficiency in these cases was confirmed biochemically. Severe neurological impairment at a young age was observed in both of these cases. In one of the reported cases of a young patient with severe coenzyme Q10 deficiency, retinopathy was demonstrated by an abnormal electroretinogram and mild pigmentary degeneration of the retina [28]. In the second case, a patient with coenzyme Q10 deficiency was diagnosed with retinitis pigmentosa at 10 years of age with concurrent optic nerve atrophy of the left eye and cataract of the right eye [29]. Sequencing of the *COQ1* gene in this latter case revealed no causative variant. Other genes in the biosynthetic pathway have been associated with other ocular abnormalities other than pigmentary retinopathy, such as such as optic atrophy (e.g., *PDSS1*/2). Why *COQ2* is the only gene thus far to definitively cause pigmentary retinopathy remains to be determined.

Attempted treatment with high dose CoQ10 supplementation in this family failed to improve the ERG signal or vision, however vision remained stable, and there was no progression of retinopathy was observed on autofluorescence or in terms of retinal atrophy on optical coherence tomography. This may suggest that CoQ10 supplementation can aid in preservation of viable retina even if there is no improvement in areas that are already affected. Thus, CoQ10 supplementation in patients with retinopathy should be considered as a possible treatment to stabilize remaining retinal function in individuals with the ocular complications associated with pathogenic *COQ2* variants. The efficaciousness of CoQ10 may be higher in cases where supplementation is started at an earlier age and prior to the onset of irreversible damage to the retina. In previously reported patients with retinitis pigmentosa stemming from variants in the *COQ2* gene, treatment with CoQ10 supplementation was not administered [24, 27]. In two reported patients with variants of the *COQ2* gene, after CoQ10 supplementation, neurologic manifestations improved significantly, but there were no noted effects on renal function [9, 19]. In one reported case, a patient with corticosteroid-resistant nephrotic syndrome and encephalomyopathy showed improvement in neurologic symptoms in response to CoQ10 supplementation, but renal function remained unchanged [20]. Response to CoQ10 supplementation seems to vary however, as seen in another case of a patient with a homozygous *COQ2* variant exhibiting renal failure [9, 18]. Twenty days after initial CoQ10 supplementation (30 mg per kg of body weight per day), this patient responded to treatment and recovered renal function [9, 18].

Variable response to supplementation by CoQ10 may also be due to variable bioavailability of CoQ10 or CoQ10 analog supplementation. Several studies have investigated the use of CoQ10 precursors that may sidestep the defects in the synthesis pathway, and such precursors may be another consideration for treatment in similar families to the study family described. One such precursor is 4-hydroxybenzoic acid (4-HBA), which is a substrate for COQ2. Although this molecule does not bypass the COQ2-mediated enzymatic step in the CoQ10 synthesis pathway, one study showed that supplementation of COQ2-deficient fibroblasts in vitro with 4-HB appeared to overcome the enzymatic defect in a dose-dependent manner, implying that certain defects may be overcome by saturating the defective COQ2 enzyme with this CoQ10 precursor [30]. Other studies have shown that intermediates further downstream of COQ2, such as β-resorcylic acid (β-RA) and vanillic acid may be used to bypass synthesis defects in the CoQ10 pathway more effectively than CoQ10 or its analogs, although these intermediates were used to address deficiencies in enzymes further down the synthesis pathway than COQ2 [31–33]. Further clinical studies will be required to assess the efficacy of these intermediates as viable therapeutic alternatives to primary CoQ10 deficiency syndromes.

## Conclusions

We present in this report a rare oculorenal syndrome in three related patients associated with novel compound heterozygous variants in the *COQ2* gene, resulting in primary coenzyme Q10 deficiency that manifests primarily as retinitis pigmentosa and renal failure. Unlike other primary CoQ10 deficiency syndromes previously described, these patients do not display neurological dysfunction at presentation. We believe this rare entity should not be missed when considering the wide differential in patients with oculorenal syndromes, as it is potentially treatable if caught early.

## Data Availability

Data sharing not applicable to this article.

## Abbreviations

CoQ10: Coenzyme q10
ES: Exome sequencing
SD-OCT: Spectral domain optical coherence tomography
ERG: Electroretinography
OD: Oculus dexter (right eye)
OS: Oculus sinister (left eye)
OU: Oculus uterque (both eyes)
ISCEV: International Society for Clinical Electrophysiology of Vision
ACMG/AMP: American College of Medical Genetics and Genomics/Association for Molecular Pathology
NS: Nephrotic syndrome
ESRD: End-stage renal disease
MSA: Multiple system atrophy
RP: Retinitis pigmentosa
